# Supplementary material for: The structure of the COPI coat determined within the cell
Source: eLife. 2017 Nov 17;6:e32493. doi: 10.7554/eLife.32493 (PMC5716667; doi:10.7554/eLife.32493)
Supplement: Transparent reporting form [file elife-32493-transrepform.docx]

***eLife’s* transparent reporting form**

We encourage authors to provide detailed information *within their submission* to facilitate the interpretation and replication of experiments. If you have any questions, please contact us: [editorial@elifesciences.org](mailto:editorial@elifesciences.org).

**Sample-size estimation**

- You should state whether an appropriate sample size was computed when the study was being designed
- You should state the statistical method of sample size computation and any required assumptions
- If no explicit power analysis was used, you should describe how you decided what sample (replicate) size (number) to use

Please outline where this information can be found within the submission (e.g., page numbers or figure legends), or explain why this information doesn’t apply to your submission:

For the EM structure determination, the largest possible sample size was used, and the resolution to which the structure can be interpreted was statistically assessed using Fourier shell correlation between independent datasets. This information is included in Materials and Methods (section “Subtomogram averaging”, pp. 12-14).

For the measurement of vesicle coat completeness, the information can be found in Materials and Methods (section “Measurement of vesicle coat completeness”, p. 15).

For morphological annotation and measurements of vesicle sizes the whole dataset of 267 vesicles and buds was used. Details can be found in Materials and Methods (section “Morphological annotation and structure variation analysis”, p. 16)

For the membrane thickness measurement in COPI buds, all buds with defined position and morphology of the donor cisterna were used. Details can be found in Materials and Methods (section “Measurement of membrane thickness in vesicles and buds”, p. 17).

For the membrane thickness measurement in Golgi cisternae all membranes perpendicular to the image plane were used. Selection, sampling, and averaging of the membranes is described in Materials and Methods (section “Measurement of membrane thickness in Golgi cisternae”, p. 17).

**Replicates**

- You should report how often each experiment was performed
- You should include a definition of biological versus technical replication
- The data obtained should be provided and sufficient information should be provided to indicate the number of independent biological and/or technical replicates
- If you encountered any outliers, you should describe how these were handled
- Criteria for exclusion/inclusion of data should be clearly stated
- High-throughput sequence data should be uploaded before submission, with a private link for reviewers provided (these are available from both GEO and ArrayExpress)

Please outline where this information can be found within the submission (e.g., page numbers or figure legends), or explain why this information doesn’t apply to your submission:

The number of tomograms and samples used for data analysis is stated in Materials and Methods (section “Cryo-electron tomography”, p. 11)

Each tomogram was acquired from a separate cell, and thus each is a biological replicate. Around five different cell cultures were used in total. Over 16 microscopy sessions, 60 tomograms were acquired that contained Golgi or coated vesicle structures. These tomograms were then sorted by image quality and tilt-series alignment precision, yielding 29 tomograms that were used for analysis.

For the structure determination, the data exclusion criteria are stated in the Materials and Methods (section “Subtomogram averaging”, p. 12-14).

For morphological annotation and structure variation analysis the number of subtomograms used and their selection criteria can be found in Fig. 4 – figure supplement 3 and in Materials and Methods (section “Morphological annotation and structure variation analysis”, p. 15).

The number of COPI buds used for membrane thickness measurement and their selection criteria are outlined in Materials and Methods (section “Measurement of membrane thickness in vesicles and buds”, p. 17).

The number of Golgi stacks and individual images used for membrane thickness measurement and their selection criteria can be found in Fig. 4H and in Materials and Methods (section “Measurement of membrane thickness in Golgi cisternae”, p. 17).

**Statistical reporting**

- Statistical analysis methods should be described and justified
- Raw data should be presented in figures whenever informative to do so (typically when N per group is less than 10)
- For each experiment, you should identify the statistical tests used, exact values of N, definitions of center, methods of multiple test correction, and dispersion and precision measures (e.g., mean, median, SD, SEM, confidence intervals; and, for the major substantive results, a measure of effect size (e.g., Pearson's r, Cohen's d)
- Report exact p-values wherever possible alongside the summary statistics and 95% confidence intervals. These should be reported for all key questions and not only when the p-value is less than 0.05.

Assessment of EM structures by Fourier shell correlation between independent half datasets is in Figure 2 – figure Supplement 1B and Figure 4 – figure supplement 3A.

Assessment of standard deviation of membrane bilayer leaflet separation in COPI structure derived by subtomogram averaging was done using bootstrapping; number of replicates and the algorithm is included in Figure 4G and in Materials and Methods.

Please outline where this information can be found within the submission (e.g., page numbers or figure legends), or explain why this information doesn’t apply to your submission:

(For large datasets, or papers with a very large number of statistical tests, you may upload a single table file with tests, Ns, etc., with reference to page numbers in the manuscript.)

**Additional data files (“source data”)**

- We encourage you to upload relevant additional data files, such as numerical data that are represented as a graph in a figure, or as a summary table
- Where provided, these should be in the most useful format, and they can be uploaded as “Source data” files linked to a main figure or table
- Include model definition files including the full list of parameters used
- Include code used for data analysis (e.g., R, MatLab)
- Avoid stating that data files are “available upon request”

Please indicate the figures or tables for which source data files have been provided:

Electron density maps (multiple figures) were deposited in the appropriate database (EMDB).
